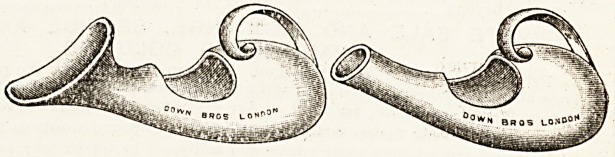# Institutional Needs

**Published:** 1913-02-22

**Authors:** 


### Institutional Needs.

THE CLEANSABLE URINAL (REGISTERED).

IN bringing out this appliance, suggested by Sister Hodnett, of West Ham Union Infirmary, Messrs. Down Bros., Ltd., of St. Thomas's Street, London, have supplied a modification on the usual pattern of vessel used in the ward and sick-room. Its simplicity and purpose are obvious from the illustration. The effectual cleansing of these receivers has always presented a difficulty, owing to the need for ensuring retention. In this model, as readers will notice, an additional opening is provided which will admit the hand or a mop for thorough cleansing.

THE "Sanitas" Company, Ltd., of Limehouse, London, E., have by Royal Warrant been appointed Disinfectant Manufacturers to His Majesty King George V.

## Figures and Tables

**Figure f1:**